# Al/SBA-15 Mesoporous Material: A Study of pH Influence over Aluminum Insertion into the Framework

**DOI:** 10.3390/nano14020208

**Published:** 2024-01-17

**Authors:** Francisco Gustavo Hayala Silveira Pinto, Vinícius Patrício da Silva Caldeira, Jhonny Villarroel-Rocha, Karim Sapag, Sibele Berenice Castellã Pergher, Anne Gabriella Dias Santos

**Affiliations:** 1 Chemistry Department, State University of Rio Grande do Norte, Mossoró 59610-210, RN, Brazil; gustavo.hayala.099@ufrn.edu.br (F.G.H.S.P.); viniciuscaldeira@uern.br (V.P.d.S.C.); annegabriella@uern.br (A.G.D.S.); 2 LABPEMOL, Institute of Chemistry, Federal University of Rio Grande do Norte, Natal 59078-970, RN, Brazil; 3 Porous Solids Laboratory, Institute of Applied Physics, CONICET, National University of San Luis, Ejército de los Andes 950, San Luis 5700, Argentina; jhonny@unsl.edu.ar (J.V.-R.); sapag@unsl.edu.ar (K.S.)

**Keywords:** Al/SBA-15, hydrothermal synthesis, pH adjustment, on-step synthesis

## Abstract

Herein, ordered mesoporous materials like SBA-15 and Al/SBA-15 were prepared using the pH adjustment method. Thus, these materials were developed in different pH of synthesis, from the pH adjustment method using a KCl/HCl solution and varying the Si/Al molar ratio (5, 25, and 75). All the ordered mesoporous materials were characterized by FRX, ^27^Al NMR, SEM, XRD, N_2_ adsorption/desorption, and CO_2_ adsorption. From the applied method, it was possible to obtain SBA-15 and Al/SBA-15 with high mesoscopic ordering based on the XRD patterns, independent of the pH employed. From the chemical composition, the insertion of higher amounts of Al into the synthesis caused a progressive improvement in the structural and textural properties of the ordered mesoporous materials. Thus, the chosen synthesis conditions can lead to different aluminum coordination (tetrahedral and octahedral), which gives these materials a greater potential to be applied. The presence of Al in high amounts provides material with the ability to form micropores. Finally, the proposed method proved to be innovative; low-cost; less aggressive to the environment, with efficient insertion of aluminum in the framework of SBA-15 mesoporous material; and practical, based on only one step.

## 1. Introduction

SBA-type materials constitute a family of molecular sieves composed of mesoporous silicas with a highly ordered pore structure, high thermal and hydrothermal stabilities, and an average pore diameter varying between 2 and 30 nm [1,2]. In this family, the material that has received the most scientific attention in the last few years has been the SBA-15 mesoporous material. The SBA-15 mesoporous silica has hexagonal ordered structures and its mesopores are interconnected through micropores, which allow for the accommodation or the diffusion of large molecules, as well as the entrance of smaller molecules through the micropores, which, in turn, gives this material wide applicability in catalysis [3,4,5] and adsorption [6,7,8,9].

The characteristics mentioned above make SBA-15 a promising material in the field of catalysis; however, due to its low number of active sites, its catalytic activity is limited [10,11,12]. Due to this fact, different heteroatoms (such as Al, Zn, and Ni, among others) are being inserted into this framework to increase its catalytic activity, with an increase in its acidity sites, improving the performance of this material [13,14,15].

Among the various molecular sieves substituted with heteroatoms, mesoporous materials with aluminum atoms incorporated into their structure possess great potential in acidity reactions for large molecules. Therefore, a large amount of effort has been dedicated to incorporating aluminum into SBA-15 through various strategies of synthesis to create acidic locations or improve its hydrothermal stability [13,16,17,18,19,20]. However, incorporating aluminum into SBA-15 is not easy. The main problem with obtaining Al/SBA-15 is the easy dissociation of the Metal-O-Si that occurs at a low pH, which complicates inserting Al (from Al^3+^ in this case) into the framework in highly acidic environments, where a large number of metallic ions are found as hydrated cations [10,16,20,21]. In addition, some of the developed methods caused a loss of the structural and textural properties of SBA-15.

Therefore, it is still necessary to deepen studies to develop new methods of synthesis with pH control, to improve the incorporation of aluminum into SBA-15′s framework. Carrying out the synthesis at a less acidic pH can overcome these difficulties. The synthesis of these materials using a pH adjustment solution could be a great alternative to overcome the problem.

The goal of the present work is to obtain an ordered mesoporous material type SBA-15 with aluminum inserted into its structure (Al/SBA-15). Due to the addition of Al increasing the acidity, the employed procedure included a pH adjustment via HCl/KCl solution with a one-step synthesis method. Such a method is simple and innovative, resulting in materials with promising properties. Additionally, the influence of the percentage of aluminum in the resultant material was verified, as well as how the aluminum is inserted into the SBA-15 framework.

## 2. Materials and Methods

### 2.1. Synthesis of Ordered Mesoporous Materials

The mesoporous materials (SBA-15 as well as Al/SBA-15) were synthesized following the patent developed by our research group [22]. The procedure consists of employing an HCl/KCl solution for pH adjustment as a scientific innovation. Two KCl/HCl solutions were prepared in an aqueous medium, with the pH adjusted to 2.0 and 3.0, respectively. In this new method, the P123 was dissolved in KCl/HCl solution, with a fixed pH of 2.0 or 3.0. Thus, the procedure started with the addition of 1.77 g of P123 (Sigma-Aldrich (St. Louis, (MO) USA)) to 44.1 mL of the KCl/HCl solution, under stirring at 1200 rpm for 2 h at 310 K (±2 K). Then, 4.37 mL of tetraethyl orthosilicate (TEOS, Sigma-Aldrich, 98% (St. Louis, (MO) USA)) was added. In this last step, for samples with Al (Al/SBA-15), the aluminum source (pseudo-boehmite, Vista, 70% Al_2_O_3_ (Brazil)) was added to the synthesis gel. Thereafter, the synthesis gel remained under stirring and heating at 308 K for 24 h. Subsequently, the synthesis gel was placed in a Teflon autoclave lined with stainless steel and heated at 373 K for 48 h. Then, the material was washed with distilled water (200 mL, the amount required to complete the removal of KCl residual), washed with 2% hydrochloric acid in ethanol, and filtered. Finally, the samples were calcined in a muffle furnace (EDG Equipamentos, FDG 3P-S 7000 (Barueri (SP) Brazil)), with a heating rate of 10 K/min until 823 K, remaining for 6 h. The ordered mesoporous materials with aluminum (Al/SBA-15) were synthesized at the molar ratios of Si/Al molar ratio = 5, 25, and 75. The stoichiometric composition established of the synthesis gel was *x* Al_2_O_3_: 1.0TEOS: 0.016P123: 0.46HCl: 127H_2_O (with *x* = 0.1, 0.02 and 0.0066 to Si/Al molar ratio = 5, 25, and 75, respectively). The Al/SBA-15 samples were coded as AS R*x* pH *y* where *x* represents the Si/Al molar ratio and *y* represents the pH of the synthesis, which was 2.0 and 3.0.

### 2.2. Characterization of Ordered Mesoporous Materials

The powder X-ray diffraction (XRD) patterns were obtained at low angles from 0.5 to 5.0° in 2*θ*. The used equipment was a Rigaku MiniFlex II model, using CuKα radiation at a voltage of 30 kV and a 15 mA tube current. First, the mesoporous parameter (*a*_0_) is obtained for each material using Equations (1) and (2). For the X-ray fluorescence (XRF) technique, the samples were placed on a polymer sample holder and placed to quantify the percentage of Al and Si. The equipment model was the Shimadzu EDX-7000.

(1) $$\lambda_{Cuk\alpha }=2\;d\left(100\right)\;sen\theta \;$$

(2) $$a_{0}\;=\frac{2\;d(100)}{\sqrt{3}}$$

The N_2_ adsorption–desorption isotherms at 77 K were performed using the Micromeritics ASAP equipment, where the samples were previously degassed at 573 K for 10 h and subsequently subjected to analysis. The specific surface area (*S*_BET_) was determined using the Brunauer–Emmett–Teller (BET) method [23] considering the IUPAC recommendations [24]. The micropore (*V*_μP-N2_) and primary mesopore (*V*_MP_) volumes were determined using the *α*_S_-plot method using the LiChrospher Si-1000 macroporous silica as reference material. The total pore volume (*V*_TP_) was obtained using the Gurvich rule [25]. The pore size distribution (PSD) was determined by applying the Villarroel–Barrera–Sapag [26] method using the adsorption branch data. The CO_2_ adsorption analyses at 273 K (up to 1000 kPa) were performed on the Micromeritics ASAP 2050 equipment. From the CO_2_ adsorption data, the narrow micropore volume (*V*_μP-CO2_) was estimated using the Dubinin–Radushkevich (DR) method and the micropore size distribution was calculated according to the Horvath–Kawazoe (HK) method for cylindrical pore geometry [27].

The morphology of the samples was determined using scanning electron microscopy (SEM) using the Shimadzu MIRA3 FERH equipment. The coordination of the aluminum atoms was determined from the nuclear magnetic resonance of Al (^27^Al MAS NMR) analysis using a Bruker AV 300 SB spectrometer (Bellerica (MA) United States) at room temperature.

## 3. Results and Discussion

Table 1 shows the data of the chemical composition of the ordered mesoporous materials, determined using X-ray fluorescence (XRF) and ^27^Al MAS NMR measurements, making it possible to calculate the Si/Al molar ratio and compare the theoretical and real values of each other.

**Figure 1:**
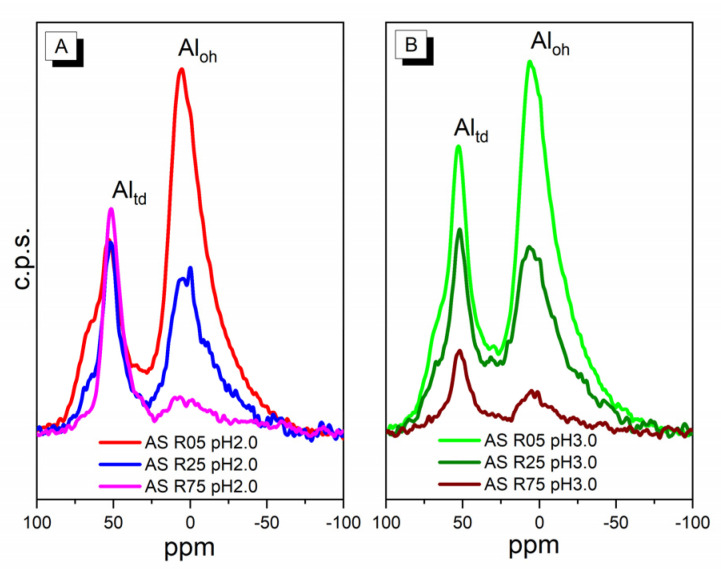
A1^27^ MAS RMN spectra of all mesoporous materials were obtained in pH 2.0 (**A**) and 3.0 (**B**) conditions and 5, 25, and 75 Si/Al ratios.

In Table 1, it is observed that the Si/Al molar ratio equal to 5, for both synthesis pHs, showed high aluminum percentages, similar among themselves and close to the theoretical value. This can be explained by a higher amount of Al available in the synthesis gel to occupy positions that otherwise would have been occupied by silicon atoms. The synthesis of AS R25, with different pH syntheses, showed values that were equivalent to the theoretical Si/Al molar ratio. However, the mesoporous materials with a Si/Al molar ratio equal to 75 showed real values that differed from the theoretical ones. Despite the amount of aluminum being similar at both pHs, this fact can be better explained by more detailed studies about the way the aluminum was inserted into the structure and the uniformity of the Al environments. To this end, ^27^Al MAS NMR measurements were performed. Figure 1 shows all of the ordered mesoporous materials with aluminum insertion, making it possible to evaluate the uniformity of the Al environments in them.

In the ^27^A1 MAS NMR measurement spectra, two peaks are observed, in which one of them, near 50 ppm, is related to the Al atom in tetrahedral coordination [13,28,29]. The tetrahedral coordination (Al_td_) implies that the Al atom is found ordered in the silica framework, thus showing that the insertion of Al during synthesis with pH control was efficient for both pHs of 2.0 and 3.0. The Al atom in tetrahedral coordination requires a charge compensation cation, which, in this case, is probably a H^+^ proton that is released from the HCl present in the pH control solution. In the ^27^A1 MAS NMR measurement spectra, the other peak near 0 ppm can be classified as the Al atom with octahedral coordination (Al_oh_), which means it is located outside the silica framework [13,28,29]. This fact may be related to the deposition of aluminum oxide on the surface of the silica. Noticeable in the ^27^A1 MAS NMR measurement spectra is the presence of more intense peaks for the materials synthesized with a lower Si/Al molar ratio, for both tetrahedral and octahedral aluminum. This fact is related to the Al amount inserted during the synthesis of the ordered mesoporous materials, which corroborates with the XRF data. Thus, the method of synthesis employed for the Al insertion was seen as promising for the insertion of heteroatoms into the silica framework in high quantities, verified using the XRF and ^27^Al MAS NMR measurements. This simple and cheap method can be applied to the effective insertion of aluminum into the SBA-15 structure, which can significantly increase the acidic properties and application possibilities. Another important point observed concerns the pH of synthesis, which directly influences the manner of insertion of the Al atom into the silica framework. Table 1 shows the relative percentage for both tetrahedral and octahedral Al coordination, calculated from the ^27^A1 MAS NMR measurement spectra. For the samples of ordered mesoporous materials synthesized at a pH of 2.0, it is observable that, however, the small Al amount inserted into the synthesis causes a direction toward tetrahedral coordination, which reaches a maximum of insertion. This can be explained by the presence of a higher quantity of charge compensation cations coming from the solution used in the synthesis gel at a lower pH. Thus, as a higher Al amount is inserted, it is directed to octahedral coordination, possibly as aluminum oxide. For the samples of ordered mesoporous materials synthesized at a pH of 3.0, the behavior of the type of aluminum atom coordination concerning the amount inserted is different. As the Al amount inserted into the synthesis increases, the proportion of aluminum also increases in both coordinations (Al_td_ and Al_oh_). However, the proportion of Al_oh_ is higher than the proportion of Al_td_ with elevated amounts of aluminum inserted. Therefore, the synthesis method at a pH of 2.0 indicates a greater efficiency in the insertion of heteroatoms. In this case, the Al atom with tetrahedral coordination is positioned in the ordered structure of SBA-15, which saturates with a low percentage of Al in the synthesis gel (Figure 1A). This is probably an important aspect to improve the catalytic properties of this material, which depend on high acidity, due to the need for compensation H^+^ in the framework.

**Figure 2:**
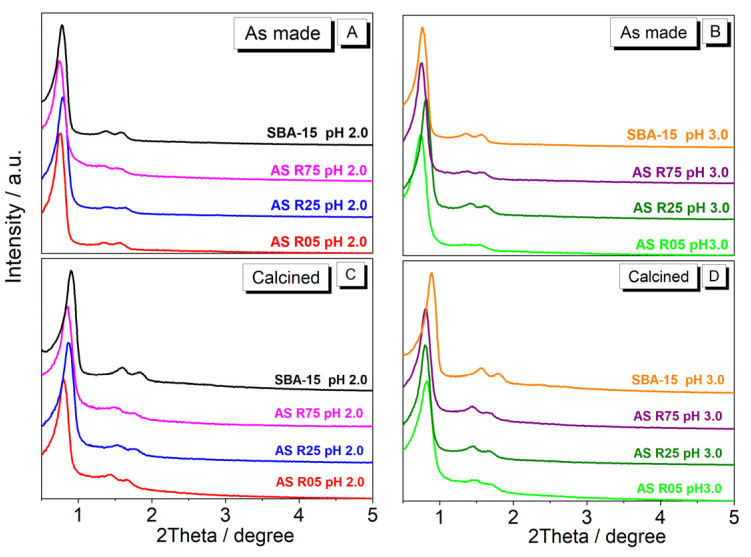
Low-angle XRD patterns of the ordered mesoporous materials (SBA-15 and Al/SBA-15) in as-made (**A**,**B**) and calcined (**C**,**D**) forms.

Figure 2A,B show the low-angle XRD patterns for the as-made samples. Thus, they prove that the synthesis method used was efficient, not only for the synthesis of the pure mesoporous materials but also for the Al insertion into the synthesis. It is possible to observe that all the XRD patterns showed high mesoscopic ordering, with (100), (110), and (200) Miller indexes, which are characteristic of SBA-15-type materials, showing the hexagonal ordered structure shape of the *P6mm* type according to the literature [1,2]. As can be observed, after calcination for the withdrawal of the organic material, the materials maintained their hexagonal structure. However, there is a slight displacement to lower values of 2θ, which could have been caused by a decrease in the interplanar spacing of the materials after calcination. This behavior was expected since calcination causes a decrease in interplanar spacing [30,31]. Some materials show different intensities in the XRD measurements. This can be explained by the difference in the pH of the medium (2.0 and 3.0) since it directly interferes with the speed and the process of micellar solvation, and this, consequently, influences the hexagonal structure of the material. Similar results relating to this pH influence on the formation of mesoporous materials were also found by Li et al. [32].

In the low-angle XRD patterns for the calcined samples (Figure 2C,D), it is possible to observe that the Al presence decreased the intensity of the reflection peaks, which indicates the structural modification of the material. In this case, the structural modification causes a decrease in the hexagonal ordering of the *P6mm* type. The Al presence in the SBA-15 structure, along with the decrease in the reflection peak intensity, causes a displacement of the peaks to lower 2θ values. Such displacement is correlated with the values of interplanar distance (*d*_(100)_), showing higher distance values between the planes. The interplanar distance values are calculated from the reflection peak using the Miller index (100) and expressed in Table 1. These values of interplanar distance do not show significant differences when compared to the procedures at different pHs. However, when comparing the materials obtained with Al insertion into the SBA-15 materials, observed is an increase in *d*_(100)_ that is not dependent on the pH employed in the synthesis. Such behavior indicates that the Al inserted into the framework promotes larger values for the interplanar distance due to the length of the Si-O-Al bonds being longer than the Si-O-Si bonds [33,34]. In the materials synthesized with a pH of 2.0, the slight decrease in the *d*_(100)_ value between the Si/Al molar ratios from 5 to 75 is possibly correlated with increasing the Al amount in octahedral coordination since the aluminum coordinated in tetrahedral form keeps constant for this pH. On the other side, in the synthesis at a pH of 3.0, the *d*_(100)_ values are kept unaltered because the increase in Al insertion is proportional both for the octahedral and tetrahedral coordination.

**Figure 3:**
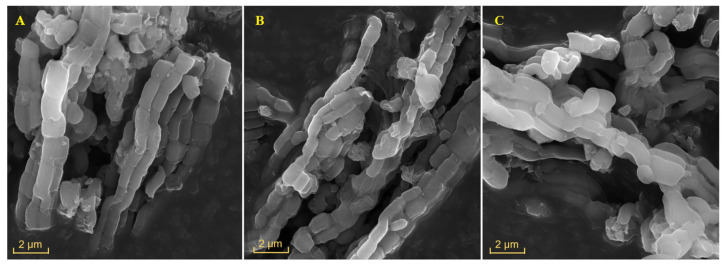
MEV micrographs of different samples: (**A**) SBA-15 pH 2.0; (**B**) SBA-15 pH 3.0; (**C**) AS R05 pH 2.0, highlighting the size of some particles.

Figure 3 shows the micrographs of SBA-15 at a pH of 2.0, SBA-15 at a pH of 3.0, and AS R05 at a pH of 2.0. The micrographs reveal materials with a morphology of particles in the form of well-organized stems, united among themselves, similar to a beaded necklace. Such morphology is characteristic of the structure of SBA-15, according to the literature [35]. This morphological observation corroborates with the low-angle XRD patterns and certifies that the synthesis method employed and the Al insertion did not modify the typical morphology of the SBA-15 materials. It was possible to obtain the values of the stems’ length when employing the *ImageJ* software (v1.54g). Values of 1.4 and 1.3 μm were obtained for the SBA-15 pH 2.0 and SBA-15 pH 3.0 materials, respectively. This suggests that the difference in pH did not show a significant difference in the condensation rate for stem growth. Therefore, it was verified that the stem lengths of the materials are similar to each other. However, comparing SBA-15 at a pH of 2.0 (1.4 μm) with AS R05 at a pH of 2.0 (1.1 μm), it is possible to see differences in the stem length, suggesting that the presence of Al promotes lower stem growth. This occurs because the presence of Al promotes more silica seeds than silica molecules, leading to a short rod morphology [36,37].

**Figure 4:**
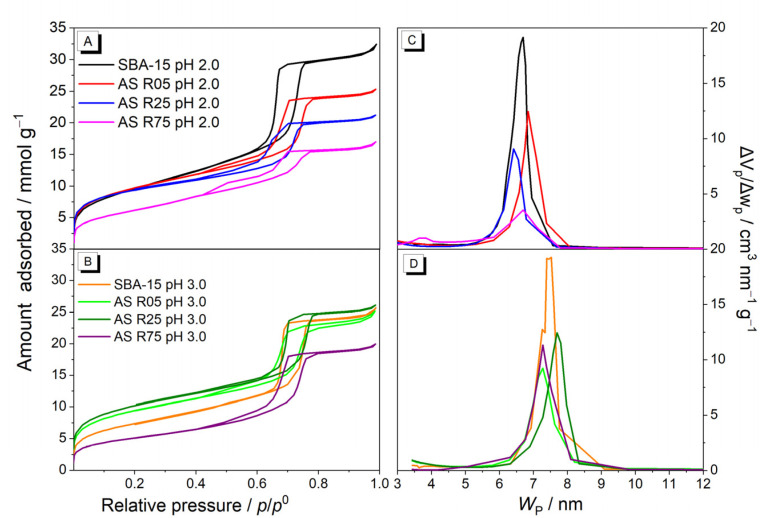
N_2_ adsorption/desorption isotherms at 77 K (**A**,**B**) and pore size distributions (**C**,**D**) of all mesoporous materials obtained with pH 2.0 and 3.0 conditions and at 5, 25, and 75 Si/Al ratios.

Figure 4 shows the N_2_ adsorption–desorption isotherms of all materials and their pore size distributions using the VBS method [26], for the materials obtained. All of the isotherms (Figure 4A,B) were classified as Type IV(a) isotherms, characteristic of mesoporous materials with capillary condensation between 0.6 and 0.8 *p*/*p*^0^ with a final plateau [25,35]. In the case of SBA-15 materials, their isotherms present a Type H1 hysteresis loop, whereas for the materials with Al insertion, mainly for those synthesized with a pH of 2.0 and also with a high Si/Al molar ratio (or less Al insertion), the profile of the hysteresis loop changes to Type H5. This fact might be related to the presence of the Al in the silica framework (Al_td_) generating a pore structure that possesses open pores and other partially closed ones [25,38], blocking micropores and reducing the specific surface area. These results are in agreement with the ^27^Al MAS NMR measurements, where the samples with a higher amount of Al atoms with octahedral coordination, which are located outside the silica framework, are not affected significantly in the mesoporous structure. Most of the materials exhibit quite vertical adsorption branches in the capillary condensation region, which is characteristic of materials with a well-defined internal pore size, according to the XRD patterns. The profile of the desorption branch in the hysteresis loop region can be related to the presence of pore blocking. The parallelism in the branches of the hysteresis loops for the AS R05 materials (both pHs of 2.0 and 3.0) shows its uniform pore size, even with a higher amount of Al incorporated, according to the XRD patterns once again, and reaffirming the impact on efficiency of the synthesis method applied. However, the presence of Al in smaller amounts provoked a decrease in the uniformity of the porous system because partially blocked mesopores were generated, which caused a shift in the hysteresis loop profile. It is noticeable that as the amount of Al in the SBA-15 decreases (5 > 25 > 75), the hysteresis loop profiles change, making these materials decrease their pore uniformity. This behavior is more accentuated in the materials synthesized with a pH of 2.0. With smaller amounts of aluminum (the AS R75 sample), there is a more prolonged hysteresis loop, leading to higher *p*/*p*^0^ intervals, which indicates a broader pore size distribution range that has been caused by the low amount of Al, according to the literature by Ungureanu et al. [12].

Table 2 shows the values of specific surface area (*S_BET_*), total pore volume (*V_TP_*), primary mesopore volume (*V_MP_*), modal pore diameter (*w_p_*), wall thickness (*e*), blocked pore volume (*V_blocked_*), and structural parameter (*a*_0_). Initially, when evaluating the SBA-15 materials synthesized at both pHs (2.0 and 3.0), a distinct behavior between them can be observed. The *S*_BET_, *V*_TP_, and *V*_MP_ values of the SBA-15 at a pH pf 3.0 are smaller when compared with the SBA-15 at a pH of 2.0 (600 vs. 790 m^2^·g^−1^, 0.87 vs. 1.10 cm^3^·g^−1^, and 0.75 vs. 0.95 cm^3^·g^−1^, respectively). This behavior can be explained by the formation mechanism type N_0_X^−^I^+^ [39], where the protonation of the PEO chains occurs, associated with the cationic surface of the silica, mediated by the negatively charged chloride counterions. When raising the pH of the medium from 2.0 to 3.0, the electrostatic interactions decrease, disfavoring the formation mechanism for the hexagonal mesoscopic ordering. However, the increase in the average pore diameter is consistent with the influence that the medium’s pH has on the formation mechanism of SBA-15. This occurs because, in more acidic mediums, the copolymer’s micelles are strongly solvated since the intensive protonation of the PEO chains reinforces the hydrophilic unit of the ethylene oxide. However, at high pH values, favorable non-polar conformation causes inductions in the PEO blocks due to the deprotonation. With this, the interior ethylene oxide units adjacent to the PPO segment become part of the hydrophobic nucleus under moderate acidity, leading to larger mesopore sizes, according to the literature by Li et al. [33].

When evaluating the insertion of the Al atom at pH values of 2.0 and 3.0 into the synthesis gel, different behaviors and effects were identified. The material with the highest percentage of inserted Al (AS R05 sample) at a pH of 2.0 demonstrates equal values for the specific surface area and modal pore diameter, lower values for the total pore volume (0.87 vs. 1.10 cm^3^·g^−1^) and primary mesopore volume (0.75 vs. 0.95 cm^3^·g^−1^), and higher values for the wall thickness (6.0 vs. 4.7 nm) when compared to SBA-15, respectively. Meanwhile, the material obtained at a pH of 3.0 (AS R05 sample) shows similar values for the total pore volume and modal pore diameter, a lower value for the primary mesopore volume (0.65 vs. 0.75 cm^3^·g^−1^), and higher values for the specific surface area (760 vs. 600 m^2^·g^−1^) and wall thickness (5.1 vs. 4.0 nm) when compared to SBA-15, respectively. For both pH values, the Al inserted interferes in the condensation process of the silanol groups for the formation of the siloxane groups (Si-O-Si) since part of the silicon atoms are substituted by the Al, forming Si-O-Al bonds, which decreases the primary mesopore volume and increases the wall thickness, corroborating with the XRD measurements. However, when comparing the materials obtained with the lowest Si/Al molar ratio, it is observed for the AS R05 at a pH of 2.0 that the strong acidic pH promoted a smaller average pore diameter and larger wall thickness when compared to the material AS R05 at a pH of 3.0. This occurs due to the influence of the pH on the formation of these materials, as discussed previously. In both cases, the condensation of the inorganic surface promoted the insertion of the Al atom in tetrahedral (Al_td_) and octahedral (Al_oh_) coordination, according to the ^27^Al MAS NMR measurements.

Analyzing the mesoporous volume of the synthesized materials not at a pH of 2.0, an increase in the mesoporous volume of the AS R75 pH 2.0 sample can be seen, until we reach the AS R05 pH 2.0 sample, which indicates that the presence of Al in high quantities promotes the formation of primary mesopores. This fact is in accordance with the ^27^Al MAS NMR measurements, which corroborates with the increase in the Al amount, where the Al_td_ remains constant while increasing the amount of Al_oh_, as already demonstrated. It can be seen that at a pH of 2.0, smaller amounts of Al inserted into the material (Si/Al molar ratios of 75) generate more blocked mesopores. On the other hand, as the amount of Al increases (Si/Al molar ratios from 5), the blocked mesopores decrease, as can be seen in Table 2, where the blocked mesopore volume decreases from 0.05 to 0.00 cm^3^·g^−1^. For materials synthesized at a pH of 3.0, the AS R75 pH 3.0 sample was the one with the lowest value of primary mesopores, and the AS R25 pH 3.0 sample showed a mesoporous volume equal to that of the SBA-15 at a pH of 3.0. This effect can be explained (with the results of ^27^Al MAS NMR) by the fact that both the amount of Al_td_ and Al_oh_ increases when increasing the amount of Al inserted into the material. Therefore, the material in R25 presents a balance between both the Al_td_ and Alo_h_ quantities, which makes it possible to obtain a material with better textural properties.

Analyzing the samples of ordered mesoporous materials obtained at a pH of 2.0, it is noticeable that the materials with the highest rate of aluminum showed more hexagonal mesoscopic organization. The larger amount of Al inserted into the synthesis probably generates a higher formation of aluminate ions, and these species can suffer an additional condensation with the silanol groups of the pre-formed mesoporous structures, leading to the formation of Si-O-Al bonds and better hexagonal mesoscopic organization. These results were similar to those obtained by Ungureanu et al. [12]. The same effect is observed for the materials synthesized at a pH of 3.0, however, in a less accentuated manner.

In Figure 4C,D, the pore size distributions show that the materials synthesized at a pH of 3.0 present values of modal pore diameter higher than the materials obtained at a pH of 2.0. Also, for both pH conditions, as the amount of Al inserted reduces, the primary mesopore volume (*V*_MP_) decreases, and the number of partially blocked mesopores increase. This fact reveals that, with high amounts of Al inserted into the SBA-15, the aluminum with octahedral coordination (Al_oh_) does not block the interior or entrance of the pores. Such disclosure strongly contributes to certifying that the synthesis method with the HCl/KCl solution, at pHs of 2.0 and 3.0, promotes a high distribution of aluminum atoms, be they in tetrahedral (Al_td_) or octahedral (Al_oh_) coordinations.

**Figure 5:**
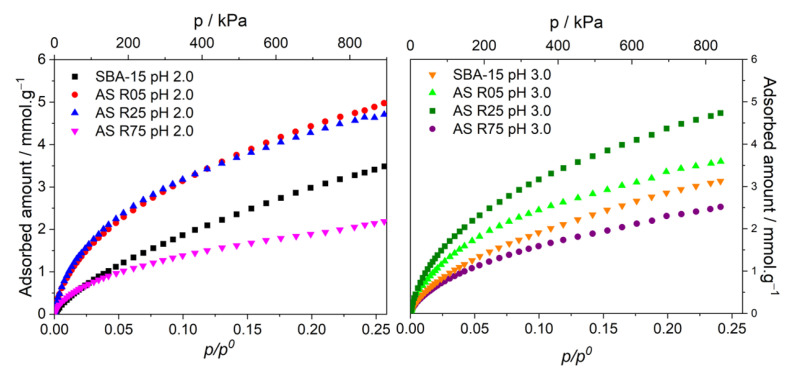
CO_2_ adsorption isotherms at 273 K of all mesoporous materials obtained in pH 2.0 and 3.0 conditions and 5, 25, and 75 Si/Al ratios.

It is known that N_2_ adsorption–desorption measurements at 77 K have a slow diffusion rate in narrow pores due to their low kinetic energy, which impairs precise analysis of the microporosity. Thus, CO_2_ adsorption experiments at 273 K, which have a higher diffusion rate, were executed to precisely evaluate the micropores present in the materials. Figure 5 shows the CO_2_ adsorption isotherms for all materials. Table 3 displays the textural properties calculated from the CO_2_ isotherm data, narrow micropore volume, and modal micropore size, with the latter calculated according to the micropore size distribution obtained using the Horvath–Kawazoe (HK) method and the micropore volume calculated using the N_2_ data for comparison with the CO_2_ data. Along with the CO_2_ isotherms and values in Table 3, as denoted, the pH did not promote significant differences in the adsorbed volume of the CO_2_ for the SBA-15 at pHs of 2.0 and 3.0, so no micropores or very few micropores were generated. The methodology and pHs at which the syntheses were carried out may have influenced the non-formation of micropores since in its original synthesis performed by Zhao [2], it was demonstrated that the material has microporous properties. In the materials with Al inserted, it was possible to observe that micropores were formed at both pHs, as can be seen in Table 3. The materials that had the smallest micropore volume were the ones with small amounts of Al inserted, in this case, the AS R75 samples. Therefore, the materials that presented the highest micropore volumes were the materials synthesized with the Si/Al ratio 25 at both pHs, as can be seen in Table 3. Probably what influences the formation of micropores is the presence of Al_oh_, considering that for samples synthesized at a pH of 2.0, the presence of Al_td_ is practically constant, and the microporous volume value for the sample AS R75 at a pH of 2.0 is 0.00 cm^3^·g^−1^. Therefore, by increasing the amount of Al_oh_ in the material, there is an increase in the number of micropores, with the highest values of micropore volume presented for the materials synthesized at the ratio of 25 (AS R25 pH 2.0 and AS R25 pH 3.0). These results are in agreement with the micropore volume data extracted from the N_2_ adsorption at 77 K. At both pHs, as the Al ratio increases, that is, the amount of Al decreases, and smaller micropores are obtained. In Table 3, the values for the modal micropore size for all the samples showed little significant difference, expect for the materials with a Si/Al molar ratio of 75 at both pHs. The SBA-15 at a pH of 2.0 exhibited the larger micropore size, followed by the materials with the highest amounts of Al. These results corroborate with the ones found using the other measurements, where the pH of the medium influences the formation mechanisms, thus generating materials with different properties depending on the pH used. In addition, the amount and coordination of the Al inserted also interfere with these properties once the condensation process of the medium is modified. Along with the microporosity evaluation of the materials obtained, it can be verified that the synthesis method with a steady pH due to the HCl/KCl solution did not affect the microporosity, demonstrated to be an efficient method.

## 4. Conclusions

The pH adjustment synthesis method using the KCl/HCl solution presented satisfactory results, since from this it was possible to obtain materials containing high amounts of Al in the SBA-15 framework. The difference in pH presented distinct behaviors for Al insertion, with a saturation of Al atoms in tetrahedral coordination during the synthesis at a pH of 2.0, and only progressive changes with the increase in Al atoms in octahedral coordination when the Si/Al molar ratio decreased. Already for materials with a pH of 3.0, the Al insertion was progressive as the amount of Al in the synthesis increased for both aluminum coordinations. Another behavior observed for the pH difference is that it interferes with the formation mechanism of the ordered mesoporous materials. Thus, the materials synthesized presented Al in two forms of coordination, which gives the ordered mesoporous materials at different ratios a wider range of applications in different processes. In addition, it was possible to insert high amounts of Al into the framework of the SBA-15, using a simple and low-cost synthesis method, without losing the highly specific area characteristics of the ordered mesoporous materials.

## Figures and Tables

**Table 1:** Percentage of silicon and aluminum presented in the ordered mesoporous materials, area of the ^27^Al MAS NMR peaks, and mesoporous parameter.

Samples	Si (%)	Al (%)	Real Ratio	Peak Area (%)		d_(100)_ (nm)
				Al_oh_	Al_td_	
				0 ppm	50 ppm	
SBA-15 pH 2.0	100	0	100	-	-	9.8
AS R05 pH 2.0	89.1	10.9	8.13	71	29	11.0
AS R25 pH 2.0	96.1	3.9	24.64	59	41	10.3
AS R75 pH 2.0	96.4	3.6	27.01	27	73	10.5
SBA-15 pH 3.0	100	0	100	-	-	10.2
AS R05 pH 3.0	88.3	11.7	7.55	63	37	10.8
AS R25 pH 3.0	96.0	4.0	24.25	61	39	11.0
AS R75 pH 3.0	96.6	3.4	27.99	43	57	11.0

Al_oh_ = Octahedral aluminum; Al_td_ = Tetrahedral aluminum.

**Table 2:** Textural properties of the materials.

Samples	S_BET_ (m^2^/g)	V_MP_ (cm^3^/g)	V_blocked_ (cm^3^/g)	V_TP_ (cm^3^/g)	a_0_ (nm)	Wp (nm)	E (nm)
SBA-15 pH 2.0	790	0.95	0.00	1.10	11.4	6.7	4.7
AS R05 pH2.0	790	0.75	0.00	0.87	12.8	6.8	6.0
AS R25 pH2.0	760	0.58	0.02	0.73	11.7	6.4	5.3
AS R75 pH2.0	515	0.50	0.05	0.58	12.1	6.7	5.4
SBA-15 pH 3.0	600	0.75	0.00	0.87	11.5	7.5	4.0
AS R05 pH3.0	760	0.65	0.00	0.86	12.4	7.3	5.1
AS R25 pH3.0	825	0.75	0.00	0.90	12.7	7.7	5.0
AS R75 pH3.0	415	0.58	0.00	0.68	12.7	8.9	3.8

*S_BET_* (m^2^/g) = specific surface area; *Vblocked* = blocked pore volume; V_TP_ (cm^3^/g) = total pore volume; *V_MP_* (cm^3^/g) = mesopore volume; a_0_ (nm) = mesoporous parameter; *Wp* (nm) = modal pore diameter; *E* (nm) = wall thickness.

**Table 3:** Microporous properties of pure SBA-15 and with Al inserted in different ratios.

Samples	*V_micro N_2__* (cm^3^/g)	*V_micro CO_2__* (cm^3^/g)	*W*p *_micro_* (nm)
SBA-15 pH 2.0	0.00	0.00	-
AS R05 pH 2.0	0.02	0.03	0.66
AS R25 pH 2.0	0.07	0.06	0.66
AS R75 pH 2.0	0.00	0.02	0.52
SBA-15 pH 3.0	0.00	0.01	-
AS R05 pH 3.0	0.04	0.05	0.75
AS R25 pH 3.0	0.05	0.05	0.72
AS R75 pH 3.0	0.01	0.02	0.50

***W*p micro** = Modal micropore size.

## Data Availability

Data are contained within the article.
